# Comprehensive evaluation of the internal and external quality control to redefine analytical quality goals

**DOI:** 10.11613/BM.2018.020710

**Published:** 2018-06-15

**Authors:** Beatriz Varela, Gonzalo Pacheco

**Affiliations:** Laboratorio de Análisis Clínicos, Quality Assurance & Quality Control Department, Montevideo, Uruguay

**Keywords:** total quality management, quality control, bias, laboratory proficiency testing/methods, clinical chemistry test

## Abstract

**Introduction:**

The aim of this work is to design a selection algorithm for total allowable error (TEa) source using a graphic tool that, by integrating internal (IQC) and external (EQC) quality control performances, enables the laboratory to evaluate which TEa source better fits the test analytical performance.

**Materials and methods:**

Two analytical performance indicators (bias and Sigma metrics) were estimated for 23 biochemistry tests during 2016. Bias was estimated on the EQC, and Sigma metrics was calculated through the results obtained in the IQC. The Sigma metrics was charted as a function of the bias (TEa%). Following the proposed algorithm (considering the hierarchy in the Milan 2014 consensus), the TEa was evaluated depending on two areas. One area in the chart was defined as the objective area in which the used TEa is the appropriate one for the analytical performance of the test under evaluation. For any test located outside this area, a performance re-evaluation was required using another source of TEa.

**Results:**

In 19 out of 23 evaluated tests, the resulting analytical performance allowed for the selection of biologic variability as TEa source. In the four remaining cases, TEa sources of lesser hierarchy were selected.

**Conclusion:**

The graphic tool designed together with the proposed algorithm enabled the laboratory to standardize the selection procedure of the most appropriate TEa for the test analytical performance.

## Introduction

Patient safety, and errors made in the healthcare system have been a very important issue in past years. Laboratory medicine services are an essential part of the delivery of healthcare; providing significant medical information vital to diagnosis, treatment and prevention of diseases. It is estimated that 60 - 70% of medical decisions are based on laboratory results (*1*, *2*).

It has been published that from the total number of errors associated to healthcare system, laboratories may participate in up to 12% (*3*). The distributional study of different errors in the medical laboratory can be done according to the different phases. Laboratory activities can be typically divided in the preanalytical, analytical and postanalytical phase. It was shown that the largest percentage of errors is found within the preanalytical phase, followed by the postanalytical phase and lastly, the analytical phase (*3*). However, according to these phases, the evaluation of the impact of such errors shows that when a negative impact is created in the patient’s health care, 52% of the time the root cause is associated to an error in the analytical phase (*4*). Therefore, the incorporation of quality assurance strategies during the analytical phase is paramount to avoid errors with a high probability of generating a negative impact in patients’ healthcare.

The management of analytical processes in a clinical laboratory involves: a) defining quality requirement based on medical usefulness; b) selecting measurement procedures and evaluating imprecision and inaccuracy to be sure the observed analytical performance satisfies the requirements; and c) selecting or designing quality control (QC) procedures based on both the quality requirements and the performance are observed for the measured procedure (*5*-*7*).

The Sigma metrics model provides an objective process to evaluate the performance of a method. This metric quantifies processes performance as a rate of defects *per* million opportunities. Sigma metrics combine bias, precision and total allowable error (TEa) and can be calculated using the formula Sigma = [(TEa – bias) / CV], where TEa is the analytical objectives (analytical specifications), and bias and CV (coefficient of variation) are the indicators of systematic and random errors, respectively (*7*, *8*).

Sigma metrics depend directly on the selected TEa. There are a great number of analytical specifications sources (TEa) found in the literature, that the laboratory faces the difficulty of having to choose the most appropriate quality specifications for use in quality planning. For this reason, when selecting TEa, it is essential to be based on relevant international references on the subject, such as the hierarchy of these specifications during the Milan consensus in 2014 (revision to the Stockholm conference in 1999) (*9*).

Based on the concepts presented hereby, the purpose of this work is to design a tool that makes the selection of analytical objectives easier, which not only refer to a known international hierarchy, but are also attainable with current technology.

## Materials and methods

### Study design

This study was carried out at LAC - Laboratorio de Análisis Clínicos, Uruguay in December 2017. LAC is a Uruguayan Clinical Laboratory accredited according to UNIT-ISO 15189:2012 standard since 2011 (*10*). In Uruguay, a laboratory that meets this standard can be accredited by the Uruguayan Accreditation Organism (OUA). A retrospective, descriptive study was performed in LAC based on the external and internal quality control performances.

The laboratory designed a selection algorithm for TEa source using a graphic tool that, by integrating internal (IQC) and external quality (EQC) control performances, enables the laboratory to evaluate which TEa source best fits the test analytical performance and to design a selection algorithm for TEa source.

### Methods

Two analytical performance indicators (bias and Sigma metrics) were estimated for 23 biochemistry tests during the same period of time, from January to December 2016. The evaluated assays were processed in a homogeneous system, analytical platform Architect ci8200 (Abbott Diagnostics, Montevideo, Uruguay), according to the manufacturer´s specifications and using proprietary reagents and reference materials as calibrators (Table 1). The following tests were included in this study: albumin (Alb), alanine aminotransferase (ALT), aspartate aminotransferase (AST), amylase (AMY), alkaline phosphatase (ALP), total bilirubin (BT), direct bilirubin (BD), calcium (Ca), cholesterol (CHOL), creatinine (CREA), gamma glutamyl-transferase (GGT), glucose (Glc), high density lipoprotein-cholesterol (HDL), iron (Fe), lactate dehydrogenase (LD), magnesium (Mg), phosphate (Phos), potassium (K), total protein (TP), sodium (Na), triglycerides (Tg), uric acid (UA) and urea.

**Table 1:** Methodological characteristics of analytes

**Analyte**	**Method**	**Calibrator list number - name**	**Calibrator´s reference material and method**
Alb	bromocresol green, colorimetric	1E65 - Multiconstituent calibrator	ERM-DA470/IFCC, gravimetric
ALT	IFCC, UV without P5P, 37 °C	None	NADH molar extinction factor/ molar extinction factor
AMY	CNPG3 substrate	None	IFCC reference procedure (2006)
ALP	p-nitrophenyl phosphate	None	IFCC reference procedure (2011)
AST	IFCC, UV without P5P, 37 °C	None	NADH molar extinction factor/molar extinction factor
TB	diazonium salt	1E66 - Bilirubin calibrator	NIST SRM 916, Doumas
DB	diazo reaction	1E66 - Bilirubin calibrator	Human samples, Doumas
Ca	arsenazo III, colorimetric	1E65 - Multiconstituent calibrator	NIST SRM 956, IDMS
CHOL	enzymatic, cholesterol oxidase/cholesterol esterase	1E65 - Multiconstituent calibrator	Abell-Kendall, volumetric
CREA	kinetic, alkaline picrate	1E65 - Multiconstituent calibrator	NIST SRM 914, IDMS
GGT	L-Gamma-glutamyl-3-carboxy-4-nitroanilide substrate	None	3-carboxy-4-nitroalanine molar extinction factor/ Molar extinction factor
Glc	hexokinase	1E65 - Multiconstituent calibrator	NIST SRM 965, IDMS
HDL	cholesterol oxidase/cholesterol esterase, colorimetric	1E68 - HDL calibrator	human samples, CDC Abell-Kendall
Fe	ferene	1E65 - Multiconstituent calibrator	NIST SRM 3126, gravimetric
LD	IFCC, UV Lactate-pyruvate	None	IFCC reference procedure (2002)
Mg	arsenazo	1E65 - Multiconstituent calibrator	NIST SRM 956, IDMS
Phos	UV, phosphomolybdate	1E65 - Multiconstituent calibrator	NIST 186-I/2186-I, gravimetric
K	ion-selective electrode	1E46 - ICT Serum calibrator	NIST SRM flame photometry
TP	biuret	1E65 - Multiconstituent calibrator	NIST SRM 927, gravimetric
Na	ion-selective electrode	1E46 - ICT Serum calibrator	NIST SRM, flame photometry
Tg	enzymatic, glycerol phosphate oxidase	1E65 - Multiconstituent calibrator	ACS grade glycerol, gravimetric
UA	uricase, colorimetric	1E65 - Multiconstituent calibrator	NIST SRM 913, gravimetric
Urea	UV, urease	1E65 - Multiconstituent calibrator	NIST SRM 912, gravimetric
ERM – European reference material. IFCC - International federation of Clinical Chemistry and Laboratory Medicine. UV- ultraviolet. P5P - pyridoxal 5`- phosphate. CNPG3 - 2-chloro-4-nitrophenyl-α-D-maltotrioside. NIST - National Institute of Standards and Technology. SRM - standard reference materials. IDMS - isotope dilution mass spectrometry. CDC - Centers for disease control and prevention. ACS - American chemical society. Alb - albumin. ALT - alanine aminotransferase. AST - aspartate aminotransferase. AMY - amylase. ALP - alkaline phosphatase. BT – bilirubin, total. BD – bilirubin, direct. Ca - calcium. CHOL - cholesterol. CREA - creatinine. GGT - glutamyl-transferase. Glc - glucose. HDL - high-density lipoprotein cholesterol. Fe - iron. LD - lactate dehydrogenase. Mg - magnesium. Phos – inorganic phosphate. K – potassium.TP - total protein. Na - sodium. Tg - triglycerides. UA - uric acid.			

Bias was first of the selected performance indicators and based on the 2016 Clinical Chemistry external quality assurance services (EQAS) BIO-RAD program results. The measured error was estimated based on the difference between the laboratory result and the best estimate of the true value (peer group mean) from each of the 12 surveys. The bias was estimated as the root mean squared of the measured error from the 12 surveys and expressed as a percentage of the total allowable error [Bias (TEa %)].

Sigma metrics was the second performance indicator selected and calculated through the results obtained in the internal quality control with an interlaboratory participation scheme (Lyphochek Assayed Chemistry Control, Level 1 and 2) corresponding to 2016 using the following formula: Sigma metrics = [TEa (%) – bias (%)] / CV (%).

Five different TEa sources were chosen to estimate the performance indicators selected by the laboratory: bias (TEa%) and Sigma metrics. Biological variation (BV), RiliBÄK (guidelines of the German medical association for the quality assurance of laboratory medical examinations), RCPA (Royal College of Pathologists of Australasia), CLIA (Clinical Laboratory Improvement Amendments) and the state of the current technology (hereinafter, BioRad EQAS Performance) estimated by the laboratory were the selected TEa sources (*11*-*14*).

The BioRad EQAS performance estimation was done through the weighted CV (%) calculation of the peer group of 12 surveys from the 2016 Clinical Chemistry EQAS BIO-RAD program. It was considered that the state of the current technology would be three times the weighted CV (*15*, *16*).

**Figure 1:**
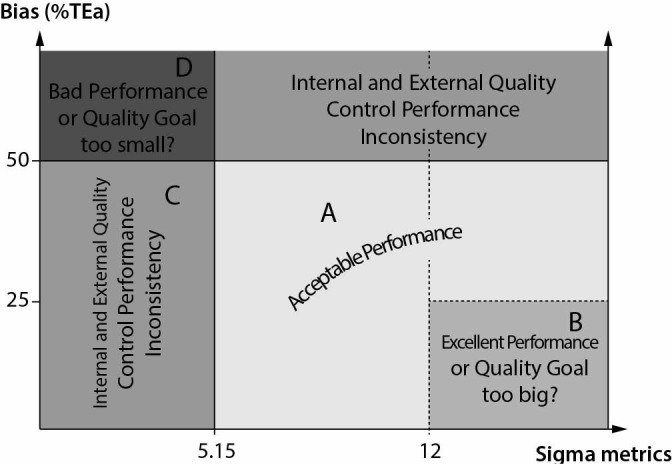
Graphic tool used to integrate the performance of external and internal quality evaluation programs. Area A: 5.15 < sigma < 12 and bias (%TEa)_2016_ < 50 or sigma >12 and 25 < bias (%TEa)_2016_ < 50. Area B: bias (%TEa)_2016_ ≤ 25% and sigma ≥ 12. Area C: sigma ≤ 5.15 and bias (%TEa)_2016_ ≤ 50% or sigma ≥ 5.15 and bias (%TEa)_2016_ ≥ 50. Area D: bias (%TEa)_2016_ > 50% and sigma < 5.15.

A graphic tool was used to integrate the performance of the external and internal quality evaluation programs in which the Sigma metrics was charted as a function of the bias (TEa%)_2016_ and four areas have been identified in the chart (Figure 1).

**Figure 2:**
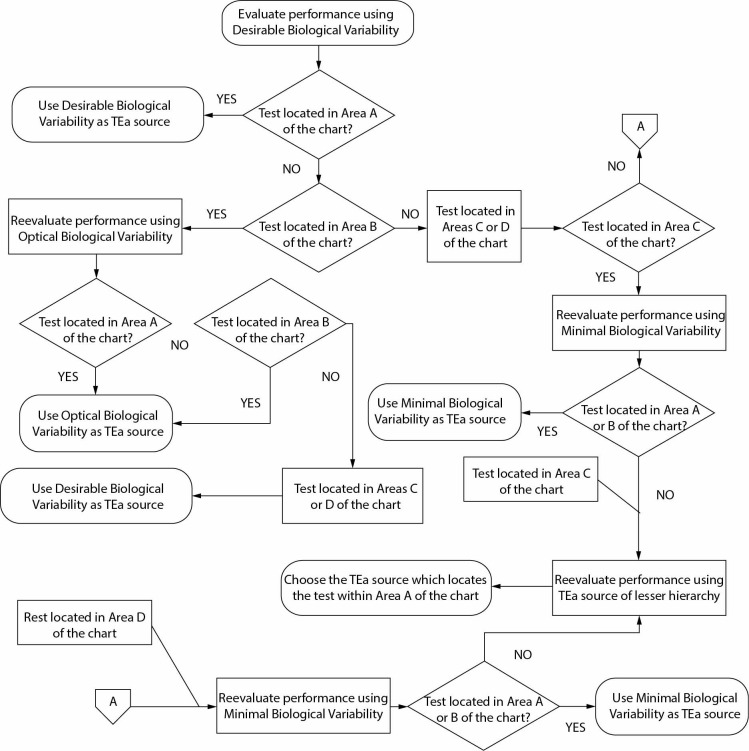
Selection algorithm for TEa source. The algorithm presented was designed on the basis of the areas defined in Figure 1.

The work algorithm designed by the laboratory to select the TEa source is summarized in Figure 2.

Alternatively, in order to prioritize the selection of the TEa source of lesser hierarchy, for the 23 evaluated tests the Sigma metrics and bias performance indicators bias (TEa%) were calculated using different TEa sources: RCPA, RiliBÄK, CLIA and BioRad EQAS performance.

The performance obtained by TEa source was related to the area in the chart in which it was located and the percentage of assays in a certain area of the chart according TEa source was calculated, which was to enable the identification of the most appropriate TEa source according to their performance in the laboratory tests. This is the TEa source that should be selected as the first option in cases where minimal biological variability could not be used.

### Statistical analysis

Twelve monthly EQAS® reports were used to calculate the bias:
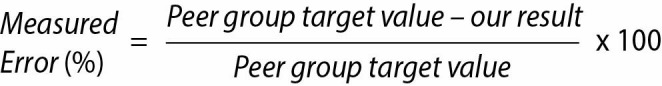


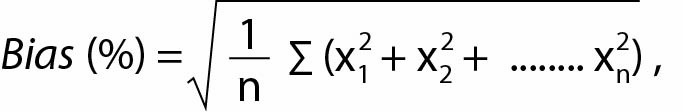
where x is the measured error (%) for each individual survey and *n* is the number of surveys. The bias was expressed as a percentage of the total allowable error, bias (TEa%), using the following formula:


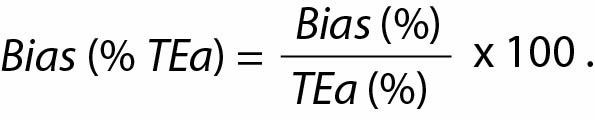


Annual Sigma metrics for each test was estimated as the average of the monthly Sigma metrics of the limiting quality control level (worst performance control level). Sigma metrics was calculated through the results obtained in the internal quality BioRad control with an interlaboratory participation scheme corresponding to 2016 using the following formula: Sigma metrics = [TEa (%) – bias (%)] / CV(%), where CV (%) was the analytical coefficient of the test method variation and the bias, the difference between the laboratory mean and the peer group. CV and bias were collected during the same time frame and the Sigma metrics was calculated monthly.

## Results

**Figure 3:**
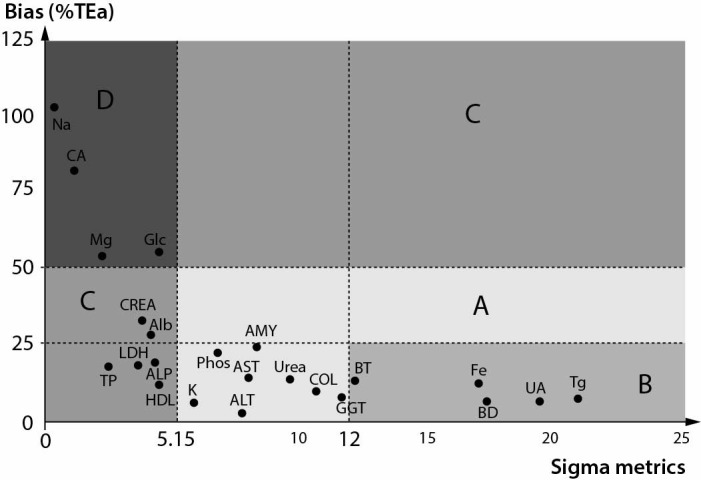
Comprehensive evaluation of the assays studied. Area A: ALT, AMY, AST, CHOL, GGT, Phos, K, Urea; Area B: BT, BD, Fe, Tg, UA; Area C: Alb, ALP, CREA, TP, HDL, LD; Area D: Ca, Glc, Mg, Na. X-axis indicates the value of Six Sigma. TEa – total allowable error. Alb - albumin. ALT - alanine aminotransferase. AST - aspartate aminotransferase. AMY - amylase. ALP - alkaline phosphatase. BT – bilirubin, total. BD – bilirubin, direct. Ca - calcium. CHOL - cholesterol. CREA - creatinine. GGT – gamma glutamyl-transferase. Glc - glucose. HDL - high density lipoprotein cholesterol. Fe - iron. LD - lactate dehydrogenase. Mg - magnesium. Phos – inorganic phosphate. K - potassium; TP - total protein. Na - sodium. Tg - triglycerides. UA - uric acid.

The main results of this work using desirable biological variability as TEa source are presented in Figure 3, in which the tests were grouped by location in the chart. From a total of 23 tests, 8 were located in area A of the chart: ALT, AMY, AST, CHOL, GGT, Phos, K, and urea. There were five tests located in the area B of the chart, BT, BD, Fe, Tg, and UA. The performance was re-evaluated for these tests using optimal biological variability as TEa source. The result obtained in this re-evaluation was that every test was located in area A of the chart. The six tests in area C of the chart (Alb, ALP, CREA, TP, HDL, and LD) were re-evaluated using minimal biological variability as TEa source. The result was that every test fell in area A of the chart except for TP which continued to be in area C of the chart. Performance of TP was re-evaluated using TEa from other sources (Table 2). The result indicated that the test was in area A of the chart for all the evaluated sources. The four tests in area D (Glc, Ca, Na and Mg) of the chart were re-evaluated using minimal biological variability as source. Only two test changed areas, Glc moved to area A and Mg, to area C. The tests that continued to be in area D were re-evaluated using TEa from other sources (RiliBÄK, RCPA, CLIA and BioRad EQAS performance), the location of the tests performance with the different sources is represented in Table 2. According to the results obtained for TP and Mg the laboratory would be able to select among the following sources: RCPA, RiliBÄK, CLIA and BioRad EQAS performance, given that any of these sources fell in area A of the chart. For Na RiliBÄK was the only TEa source that made the test be located in area A, and for Ca the laboratory would be able to select among the following sources: RiliBÄK, CLIA and state of the art.

**Table 2:** Performance indicators according to selected TEa source

	**Bias (TEa, %)**				**Sigma Metrics**			
**Analyte**	**RCPA**	**RiliBÄK**	**CLIA**	**Biorad EQAS performance**	**RCPA**	**RiliBÄK**	**CLIA**	**Biorad EQAS performance**
TP	13.0	6.5	6.5	8.9	6.28	11.1	11.1	7.5
Ca	52.3	20.9	28.2	33.7	2.9	9.2	9.3	5.1
Mg	32.3	17.4	10.4	21.4	5.7	8.2	14.3	6.7
Na	37.6	15.0	28.2	19.3	2.6	6.4	3.6	4.8
RCPA - Royal College of Pathologists of Australasia. RiliBÄK - guidelines of the German medical association for the quality assurance of laboratory medical examinations.. CLIA - Clinical Laboratory Improvement Amendments. TP - total protein. Ca - calcium. Mg – magnesium. Na – sodium.								

In 19 out of 23 evaluated tests the analytical performance allowed for the selection of biologic variability as TEa source.

According to the results presented in Table 3 and 4, the TEa source that best adjusts itself to tests performance in the laboratory is RiliBÄK because the performance indicators of 19 of the evaluated tests with this source fall into area A or B of the chart. RiliBÄK as a source of TEa are not available for AMY, BD, HDL and Fe.

**Table 3:** Analyte location in the chart according to the evaluated performance with different TEa sources

**Analyte**	**RCPA**	**RiliBÄK**	**CLIA**	**Biorad EQAS Performance**
Alb	Area A	Area B	Area A	Area A
ALT	Area C	Area A	Area A	Area C
AMY	Area A	NA*	Area B	Area A
ALP	Area A	Area A	Area B	Area C
AST	Area A	Area A	Area A	Area C
BT	Area A	Area A	Area B	Area A
BD	Area A	NA*	NA*	Area C
Ca	Area D	Area A	Area A	Area C
CHOL	Area A	Area B	Area B	Area C
CREA	Area C	Area A	Area A	Area C
GGT	Area C	Area A	NA*	Area A
Glc	Area A	Area A	Area A	Area A
HDL	Area C	NA*	Area B	Area A
Fe	Area A	NA*	Area A	Area C
LD	Area C	Area A	Area A	Area C
Mg	Area A	Area A	Area B	Area A
Phos	Area C	Area B	NA*	Area C
K	Area A	Area A	Area A	Area C
TP	Area A	Area A	Area A	Area A
Na	Area C	Area A	Area C	Area C
Tg	Area B	Area B	Area B	Area A
UA	Area B	Area B	Area B	Area A
Urea	Area A	Area B	Area A	Area C
*NA – source not available (30,32). RCPA - Royal College of Pathologists of Australasia. RiliBÄK - guidelines of the German medical association for the quality assurance of laboratory medical examinations. CLIA - Clinical Laboratory Improvement Amendments. Alb - albumin. ALT - alanine aminotransferase. AST - aspartate aminotransferase. AMY - amylase. ALP - alkaline phosphatase. BT – bilirubin, total. BD – bilirubin, direct. Ca - calcium. CHOL - cholesterol. CREA - creatinine. GGT – gamma glutamyl-transferase. Glc - glucose. HDL - high density lipoprotein cholesterol. Fe - iron. LD - lactate dehydrogenase. Mg - magnesium. Phos – inorganic phosphate. K - potassium; TP - total protein. Na - sodium. Tg - triglycerides. UA - uric acid.				

**Table 4:** Distribution of the assays’ location in the chart according to TEa source expressed in percentage

**TEa source**	**Area A**	**Area B**	**Area C**	**Area D**
RCPA	56.5%	8.7%	30.4%	4.4%
RiliBÄK	68.4%	31.6%	0.0%	0.0%
CLIA	55.0%	40.0.%	5.0%	0.0%
Biorad EQAS performance	43.5%	0.0%	56.5%	0.0%
TEa – total allowable error. RCPA - Royal College of Pathologists of Australasia. RiliBÄK - guidelines of the German medical association for the quality assurance of laboratory medical examinations. CLIA - Clinical Laboratory Improvement Amendments. EQAS - external quality assurance services. TP- total protein. Ca-calcium. Mg – magnesium. Na – sodium.				

## Discussion

The model designed by our laboratory presents three main strengths: 1) it holds the ability of integrating internal and external quality control performances, which are two paramount tools for the evaluation of the analytical system stability throughout time; 2) it allows the evaluation of the analytical performances in relation to the selected performance specifications; 3) it works as a tool for the alignment of analytical performance specifications in accordance to the hierarchical criteria exposed in the 2014 Milan consensus (*9*).

Internal quality control as an analytical performance indicator allows for the estimation of precision in the evaluated concentrations. This is added to the possibility of estimating the bias by using third opinion materials with robust even groups (*15*). These characteristics make it a good tool to evaluate each of the error components (systematic and random) separately and/or comprehensively through Sigma metrics.

External quality control as an analytical performance indicator is complementary to internal control. It allows the evaluation of measurement procedures in concentrations that are not evaluated by the latter. Likewise, error quantification (measurement error) is used as a way of evaluating the measuring precision of assays, because the result of each survey is effected by both error components (systematic and random) being an expression of these integration. Moreover, the analysis of a group of surveys during a long period of time in which the analytical system has been stable allows for the estimation of another performance indicator as the measuring bias.

For the performance evaluation using said tools, it is highly important to define a margin of error. The level of performance required to facilitate clinical decision-making has been given a number of names. The currently most widely applied terms are quality specifications, quality goals, analytical performance goals and total allowable error (*16*). The TEa is the amount of error that can be tolerated without invalidating the medical usefulness of the analytical result. TEa is used to define acceptable analytical performance for assessment of an individual instrument’s analytical performance, quality control validation and as a measure of agreement or comparability of results for analytes measured on different systems (*17*).

In a 2013 review on improvements in quality and patient safety, Plebani emphasized the need for further improvements in analytical quality: “A better analytical quality should be achieved by setting and implementing evidence-based analytical quality specifications in everyday practice; if this will be done, rules for internal quality control and external quality assessment procedures would be more appropriate. Moreover, there is a compelling need for standardization programs aimed at improving metrological traceability and correcting biases and systematic errors. Finally, more stringent metrics, such as Six Sigma, should be largely introduced in clinical laboratories, to further improve current analytical quality.” (*18*).

Due to the clinical impact that a wrongful selection of quality requirements may have and the great amount of analytical decisions that are made based upon it, it is paramount that laboratories establish clear procedures to align said selection.

The proposed TEa selection algorithm is presented as a possible solution to this problem. The flexibility of the associated quality specifications based on biological variability allows having a wider range of allowable error to be selected for each assay without losing the TEa rank in hierarchy. One of the difficulties covered by the model is how to proceed when the specifications based on biologic variability are unattainable. In these cases, the algorithm proposes the selection of sources of lesser hierarchy (third level in the 2014 Milan consensus). When this happens due to the great number of available sources, the laboratory is faced with selecting specifications that depend on the offered features by the industry in which there is an unclear relationship between the specification adaptation and the clinical utility. Implicitly, when a TEa of this level is selected, we are establishing a minimum performance specification. This is linked to the state of technology in a certain analytical context.

Along these lines, the proposed method covers the main requirements based on the schemes of external quality evaluation as well as regulations linked to obtaining performances used in the analytical platform. The aim of this is to standardize the selection of a third level of hierarchy requirement against the others, in agreement with its adaptation to the technical features attained by an analytical system stable in time. It is worth noting too that the evaluated measuring procedures are accredited by Standard UNIT ISO 15189:2012.

We cannot fail to mention that there are cases in which the analytical specifications based even in the optimal level of biologic variability highly exceed those estimated through the state of current technology. For example, for BD, the optimal specification is 22.3%, whiles the estimated specification through BioRad EQAS performance in 2017, was 12.7%. In these cases, the laboratory can decide to use a requirement of lesser hierarchy (level 3) in which the allowable error is lower adapting to the analytical system used and decreasing the amount of variation added to true test result variability.

The obtained results show a correct suitability for the proposed algorithm, its implementation being possible in the evaluated analytical platform. In addition, Sigma metrics was the performance indicator that ended up defining the TEa in all cases. No assays were found in which the analytical performance estimated through the internal quality control (Sigma metrics) had a better performance than the estimated through the external quality control [Bias (TEa%)]. This can be explained by the different factors that can be considered extra-analytical inherent to the manipulation of the internal quality control material (*i.e.* storage, aliquotation, freezing, analytes stability, multiple daily procedures, *etc.*) while the impact of these factors in the external evaluation programs is significantly lower or non-existent (depending on the case). Therefore, it can be said that the Sigma metrics would be underestimated in this model, as the variables used do not affect the decisions made about patients’ samples.

The Sigma metrics values are useful in setting the internal quality control acceptability criteria and control strategy based on the Westgard rules (*19*-*21*). For analytes attaining sigma below 5.15, a multi-rules procedure has to be used and/or quality control should be run at a higher frequency to improve the probability of error detection. This fact, added to the Sigma metrics underestimation, implies a questionable rise in the complexity of the quality control system regarding daily operation of control material procedures and evaluation of the results. It is for this reason that it is decided to evaluate the performance indicators using TEa sources of lesser hierarchy that allow to locate the assay in Area A of the chart, which is associated to rules of simple control with high probability of error detection and low probability of false rejection.

The decision to choose requirements of lesser hierarchy was also because the only assays that presented a critical sigma performance lower to three using minimal biologic variability as TEa source were Na and CA. This coincides with how demanding this requirement is, which makes it unattainable due to the current state of technology. The quality specification for minimal biologic variability is of 1.1% and 3.6% for Na and Ca, respectively. Assuming a hypothetical scenario where there is no bias, CV should be lower than 0.21% for Na and lower than 0.69% for Ca to reach a sigma performance higher than 5.15 which makes it a difficult requirement to attain currently. For this reason, a quality requirement of lesser hierarchy had to be selected for these two cases.

For 15 out of the 23 studied assays, the selected quality requirement of biologic variability was higher than 10%. This quality requirement is more permissive the assay tolerates a greater imprecision and bias without modifying its location in the chart.

In six assays (Alb, UA, CHOL, K, Mg and TP), the selected quality requirement of biologic variability was within 5.4% and 10%. Only for Mg and TP biologic variability could not be selected as quality requirement. The minimal quality requirements of biologic variability are 5.4% and 7.2% for TP and Mg, respectively. Considering the zero-bias model, CV should be lower than 1.05% for TP and lower than 1.40% for Mg to reach a sigma performance higher than 5.15 which makes it a difficult requirement to attain. For this reason, a quality requirement of lesser hierarchy had to be selected for these two cases.

In regards to the selection of the TEa for its use as an specification of analytical performance, the results obtained by the laboratory, in this work, are consistent with those obtained in other published studies where it is concluded that biological variability represents, in many cases, a very difficult goal to achieve (*i.e.* Na, Ca, *etc*.) (*22*-*25*). For these assays the selection and use of TEa source of lesser hierarchy are essential, and this element should be considered as an input in any algorithm designed to decide which TEa goal is most appropriate for it.

The algorithm developed by the laboratory seeks to combine the concepts of hierarchy and demand. Stablishing biological variability as an initial goal for being the one with the highest hierarchy and also, usually, the most demanding. The idea implicit in this decision lies in generating a tool aligned to the continuous improvement of the processes, where the purpose is to identify in which assays the analytical goals could be attainable using more stringent quality goals such as biological variability instead of using less demanding goals that would allow too much tolerance of the processes, even when they are regulatory. At the same time, this algorithm allows to identify TEa goals that are too demanding for analytical performance for some typical field assays. At this point, it is worth mentioning that in those cases where there are regulatory requirements (mandatories, for instance CLIA) the laboratory should always verify that the requirements selected, following the proposed algorithm, are more stringent than those established as mandatory by the corresponding body.

The laboratory was faced with immense difficulty to apply this model due to the absence of IT tools that enabled the integration of performance indicators associated to the internal and external quality control results. The existence of such an IT tool would significantly simplify the possibility of implementing this model to laboratories, which would enable the standardization of the selection procedure for TEa.

The model designed by our laboratory presents some limitations which occasionally restrict its use. It must be considered that to estimate the bias the target value was considered as the best estimation of the true value defined by the value of the even group. It is also worth noting, there might be better estimations of the true value such as those values obtained through the analysis completed done over materials quantified by a reference method (reference materials). Furthermore, the model cannot be applied when a first opinion internal quality control is being used as peer group values are not known in these cases. This prevents the calculation of the bias and thus, the Sigma metrics. The usage of non-accredited external quality evaluation programs under Standard ISO 17043 does not ensure statistical robustness for assigning the best estimation of the true value. Lastly, the model designed for the selection of the TEa has the limitation that the biologic variability is not available for all analytes.

In conclusion, the graphic tool designed together with the proposed algorithm enabled the laboratory to standardize the selection procedure of the most appropriate TEa for the test analytical performance.
